# Herpes Simplex Esophagitis in Immunocompetent Host: A Case Report

**DOI:** 10.1155/2009/717183

**Published:** 2009-09-09

**Authors:** G. Geraci, F. Pisello, G. Modica, F. Li Volsi, M. Cajozzo, C. Sciumè

**Affiliations:** Section of General and Thoracic Surgery, University Hospital of Palermo, 90100 Palermo, Italy

## Abstract

*Introduction.* Herpes simplex esophagitis is well recognized in immunosuppressed subjects, but it is infrequent in immunocompetent patients. We present a case of HSE in a 53-year-old healthy man. *Materials and Methods.* The patient was admitted with dysphagia, odynophagia, and retrosternal chest pain. An esophagogastroduodenoscopy revealed minute erosive area in distal esophagus and biopsies confirmed esophagitis and findings characteristic of Herpes Simplex Virus infection. *Results.* The patients was treated with high dose of protonpump inhibitor, sucralfate, and acyclovir, orally, with rapid resolution of symptoms. *Discussion.* HSV type I is the second most common cause of infectious esophagitis. The majority of symptomatic immunocompetent patients with HSE will present with an acute onset of esophagitis. Endoscopic biopsies from the ulcer edges should be obtained for both histopathology and viral culture. In immunocompetent host, HSE is generally a self-limited condition. *Conclusions.* HSE should be suspected in case of esophagitis without evident cause, even if the patient is immunocompetent. When the diagnosis of HSE is confirmed, careful history and assessment for an immune disorder such as HIV infection is crucial, to look for underlying immune deficiency.

## 1. Introduction

Herpes simplex virus (HSV) has been recognized with increased frequency as an opportunistic invader of the esophagus in immunosuppressed, immunocompromised, or severely ill subjects (reactivation or primary infection).

Herpes simples esophagitis (HSE) may occasionally occur in otherwise healthy and immunocompetent patients who had no underlying immunologic problems [1, 2].

We herein present a case of immunocompetent adult with HSE who had a dramatic response to acyclovir therapy.

## 2. Case Report

A 58-year-old man presented to our endoscopic unit for 1 month history of moderate dysphagia for liquids and solids, odynophagia, and retrosternal chest pain.

He had no significant medical or surgical history, and he denied abuse of tobacco or alcohol.

Clinical history and physical examination were unremarkable.

**Figure 1:**
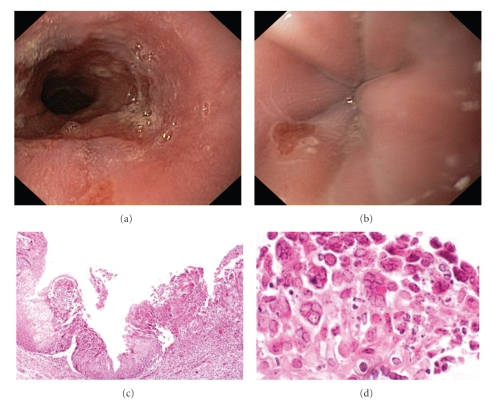
(a) Endoscopic (b) and histological ((c) [×100], (d) [600×]) appearance.

Esophagogastroduodenoscopy (EGD) was performed and revealed small erosion (6 mm) in the distal esophagus (Figures 1(a)  and 1(b)). The rest of the endoscopic examination was normal. Multiple esophageal biopsies were obtained and sent for histopathologic examination.

Histopathology from the esophageal biopsy specimens was consistent acute, severe inflammation, multinucleated giant cells with nuclear molding and nuclear chromatin with a groundglass appearance, findings characteristic of herpes virus infection (Papanicolaou's stain, orig. mag. ×100 and ×600), typical of HSV type 1 infection (Figures 1(c)  and 1(d)). Subsequent polymerase chain reaction (PCR) confirmed the diagnosis of HSE.

After diagnostic confirmation and negativity of HIV testing, the patients started treatment with high dose of proton-pump inhibitor (80 mg a day), sucralfate, and acyclovir 800 mg daily for 5 days, orally, with rapid resolution of symptoms and complete “restitutio ad integrum” at EGD.

However, he had no risk factors for HIV infection and no personal history of HSV infection or Herpes Zoster reactivation. 1-year followup clinical and at EGD is negative.

## 3. Discussion

After candidiasis, HSV type I is the second most common cause of infectious esophagitis. HSE is most commonly seen in immunocompromised patients with AIDS (1% of all patients with AIDS and 1.8%–4.3% of patients with AIDS at autopsy) [3], an underlying malignancy, a debilitating illness in patients who have been treated with radiation, steroids, or antiblastic chemotherapy.

HSE in immunocompetent hosts is rare and it may represent either a primary disease or reactivation of a latent infection. 

Typically, the patient is a young (<40 years in 78% of cases), healthy male who presents with acute odynophagia, dysphagia, or heartburn, with or without prodromal symptoms (fever, pharyngitis, respiratory symptoms) or oral lesions. Prior exposure to a family member with possible HSV lesions has been reported in about 20% of cases [2, 4].

Most symptomatic immunocompetent patients with HSE will present with an acute onset of esophageal complaints, but a subset of patients (24%) will present with a prodrome of symptoms, including odynophagia (76% of patients), fever (44%–63%), and respiratory manifestations (sore throath in 23%). Other common complaints associated with HSE may include retrosternal pain (60%), heartburn (50%), dysphagia (21%), myalgia (21%), and poor oral intake (13%) [2] (Table 1).

Oral manifestations (Herpes labialis) may precede the onset of odynophagia by 1–4 days, coincide with it or develop 1–5 days afterward [2].

HSE has characteristic endoscopic appearances (the distal or mid-esophagus is commonly involved in more than 50% of cases, although in 26% of cases the entire esophagus is affected) [2]: in the early stage, vesicles are seen, which then slough to form discrete, circumscribed ulcers with raised edges; the mucosa is friable (84%). These lesions have punched-out or volcano-like appearances. Cobblestoning can be seen due to clustering of these lesions. Exudate is present in a majority of cases [5]. Mucosal necrosis is seen in the late stage [6] (Table 2).

In case of esophageal erosions or if HSE is clinically suspected, biopsies from the ulcer edges should be obtained for both histopathology and viral culture [7]. Virus isolation by cell culture has traditionally been considered the diagnostic “gold standard” for HSV infection.

The characteristic histologic appearance is the presence of multinucleated giant cells with eosinophilic intranuclear inclusions, called Cowdry type A intranuclear inclusions and nuclear chromatin with a groundglass appearance.

In recent years, HSV DNA PCR is considered the most sensitive, cost effective, rapid, and easiest diagnostic tool of HSV infection [8].

Serology is of limited value as a majority of healthy individuals will have a prior exposure to HSV, unless there is seroconversion.

Antiviral therapy, in the early stage, is advisable to hasten recovery and to rapidly achieve symptomatic relief [9]; treatment with the nucleoside analog acyclovir has been shown to be effective for HSE. The duration of illness ranges from 4 to 9 days in those receiving antiviral therapy compared with 10 to 17 days in those receiving symptomatic treatment alone. Although HSE is noted to be a self-limited condition, cases of immunocompetent patients experiencing significant complications, including gastrointestinal bleeding and esophageal perforation, have been reported [10]. Recurrence of this condition is rare.

## 4. Conclusions

HSE should be suspected in otherwise healthy subjects (irrespective of age) with symptoms (odynophagia, heartburn) suggestive of esophagitis without obvious cause, particularly in patients with ulcerations in distal or mid-distal esophagus at endoscopy. In these cases, endoscopic biopsy from the edge of ulcers should be examined microscopically and submitted for viral culture.

When HSE is incountered, a careful history and assessment for an immune disorder such as HIV infection is crucial, to look for possible underlying pathology.

HSE in the immunocompetent host is self-limiting, but antiviral therapy may shorten the illness if started early.

## Figures and Tables

**Table 1:** Symptoms [2].

Acute onset of esophagitis without prodrome	*Retrosternal pain*	**76**%
	*dysphagia*	
	*heartburn*	
Prodrome		**24**%
	*Odynophagia*	*76*%
	*Fever*	*44*%–*63*%
	*Sore throath*	*23*%
	*Myalgia*	*21*%
	*Poor oral intake*	*13*%

**Table 2:** Endoscopic appearance [2, 5, 6].

Localization	*Distal or mid-esophagus*	>50%
	*Entire esophagus*	26%
Early stage	*Exudate*	90%
	*Friable mucosa*	84%
	*Vesicles*	80%
	*Circumscribed ulcers*	40%
	*Cobblestoning (lesions confluence)*	30%

Late stage	*Necrosis*	40%

## References

[1] Rongkavilit C, El-Baba MF, Poulik J, Asmar BI (2004). Herpes simplex virus type 1 esophagitis in an immunocompetent adolescent. *Digestive Diseases and Sciences*.

[2] Ramanathan J, Rammouni M, Baran J, Khatib R (2000). Herpes simplex virus esophagitis in the immunocompetent host: an overview. *American Journal of Gastroenterology*.

[3] Buss DH, Scharyj M (1979). Herpesvirus infection of the esophagus and other visceral organs in adults. Incidence and clinical significance. *American Journal of Medicine*.

[4] Galbraith JC, Shafran SD (1992). Herpes simplex esophagitis in the immunocompetent patient: report of four cases and review. *Clinical Infectious Diseases*.

[5] Solammadevi SV, Patwardhan R (1982). Herpes esophagitis. *American Journal of Gastroenterology*.

[6] McDonald GB, Sharma P, Hackman RC, Meyers JD, Thomas ED (1985). Esophageal infections in immunosuppressed patients after marrow transplantation. *Gastroenterology*.

[7] Klotz DA, Silverman L (1974). Herpes virus esophagitis, consistent with herpes simplex, visualized endoscopically. *Gastrointestinal Endoscopy*.

[8] Slomka MJ, Emery L, Munday PE, Moulsdale M, Brown DW (1998). A comparison of PCR with virus isolation and direct antigen detection for diagnosis and typing of genital herpes. *Journal of Medical Virology*.

[9] Lee B, Caddy G (2007). A rare cause of dysphagia: herpes simplex esophagitis. *World Journal of Gastroenterology*.

[10] Kurahara K, Aoyagi K, Nakamura S (1998). Treatment of herpes simplex esophagitis in an immunocompetent patient with intravenous acyclovir: a case report and review of the literature. *American Journal of Gastroenterology*.

